# Topological phase transition between a normal insulator and a topological metal state in a quasi-one-dimensional system

**DOI:** 10.1038/s41598-021-92390-x

**Published:** 2021-06-21

**Authors:** Milad Jangjan, Mir Vahid Hosseini

**Affiliations:** Department of Physics, Faculty of Science, University of Zanjan, Zanjan, 45371-38791 Iran

**Keywords:** Physics, Condensed-matter physics, Topological matter

## Abstract

We theoretically report the finding of a new kind of topological phase transition between a normal insulator and a topological metal state where the closing-reopening of bandgap is accompanied by passing the Fermi level through an additional band. The resulting nontrivial topological metal phase is characterized by stable zero-energy localized edge states that exist within the full gapless bulk states. Such states living on a quasi-one-dimensional system with three sublattices per unit cell are protected by hidden inversion symmetry. While other required symmetries such as chiral, particle-hole, or full inversion symmetry are absent in the system.

## Introduction

In recent years, the exploring of topological phases has been at the center of attention, in particular, in condensed matter systems^1^. The key feature is the emergence of symmetry protected gapless boundary modes due to topological bulk states. Topological phases mainly have been categorized into topological insulators^2–5^ and topological superconductors^6^ that are studied theoretically and experimentally. In contrast to these topological phases where edge states reside within the gap of bulk states, there are other types of unconventional topological phases known as topological semi-metals^7^ and topological metals (TMs)^8,9^. In the former, there exist band touching nodes at Fermi energy occurring in three-dimensional noncentrosymmetric or magnetic materials. While the latter case, which can also take place in low-dimensional systems, has a finite Fermi surface.

Depending on the properties of edge states, topological semimetals and metals can be regarded as two types. First, while topological edge states remain isolated in the bandgap, Fermi level crosses bulk and edge states at different momenta (quantum numbers). This situation, for instance, has been reported in TMs^8^, semimetals^10^, and even in a narrow energy window of topological insulators^11^. Second, gapless edge states coexist with gapless bulk states such that some edge and bulk states would have not only the same energy but also the same momentum (quantum number). This phase has been investigated in a quasi-one-dimensional (1D) system^18^ where the finite-energy edge states can penetrate into bulk states hybridizing with them^12^ and in a 1D system where the coexistence of edge and bulk states occurs at Fermi energy in a single point of parameter space^9^. However, this kind of TM phase deserves to be investigated further with a more stable feature.

Often, on the other hand, topological superconducting or insulating phase can be settled down, respectively, on trivial superconductors or insulators through occurring topological phase transition when a band inversion takes place. Furthermore, in previous works, it has been shown that the TM phase can be established on a trivial metallic ground state through closing-reopening of subband gap^9^ or main bandgap^8^. So, it is indeed intriguing to have a situation in which the underlying states in the nontrivial and trivial topological phase of a system belonging to different states can be related to each other, possibly, with a new kind of topological phase transition.

**Figure 1:**
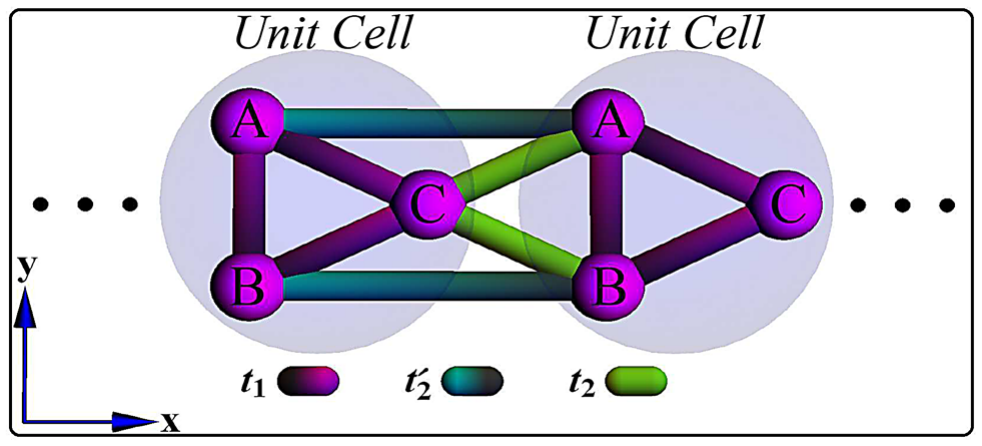
(Color online) Schematic geometry of quasi-1D lattice comprising of three sublattices A, B, and C per unit cell. Intra and horizontal (oblique) inter unit cell hoppings are indicated by dark magenta and dark cyan (green) colors, respectively.

**Figure 2:**
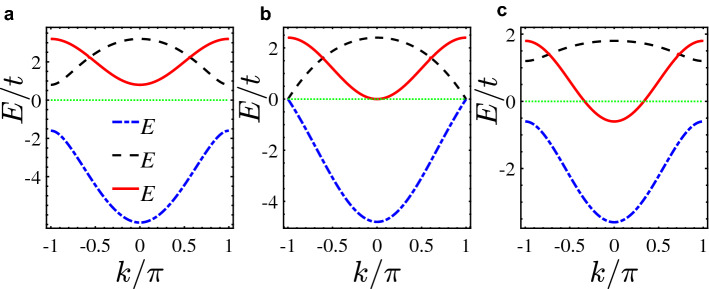
(Color online) Band structure of the system for (**a**) $t_1=-2t$, (**b**) $t_1=-1.2t$, and (**c**) $t_1=-0.6t$ under periodic boundary conditions. Horizontal thin line indicates Fermi level. Here, $t^{\prime }_2=t_2/2=-0.6t$.

**Figure 3:**
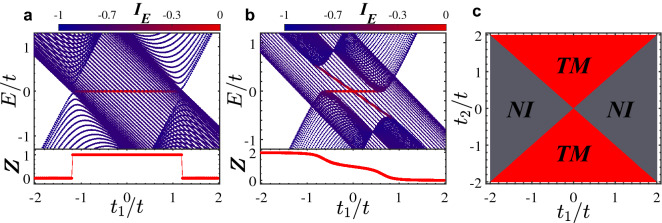
(Color online) Energy spectra and the inverse participation ratio with their relevant topological invariant as a function of $t_1/t$ under open boundary conditions for (**a**) $t^{\prime }_2=t_2/2$ and (**b**) $t^{\prime }_2\ne t_2/2=-0.3$. Here, $t^{\prime }_2=-0.6t$. (**c**) Topological phase diagram as functions of intra and inter unit cell hoppings $t_1$ and $t_2$. The red region represents the nontrivial TM phase while the gray region indicates NI.

In this paper, within the tight-binding approach in a quasi-1D model with three sublattices per unit cell (Fig. 1), the occurrence of a new kind of topological phase transition between a normal insulator (NI) and TM is investigated. Interestingly, in such phase transition, in addition to gap closing-reopening between two bands, another band passes the Fermi level (Fig. 2) resulting in the emergence of zero-energy edge states within bulk states (Fig. 3a). Although the system has no explicit symmetry, in a subspace of the Hilbert space, there is a hidden inversion symmetry protecting the TM phase.

## Model and theory

The model we consider is a three-component quasi-1D lattice as represented in Fig. 1. Using the tight-binding theory, the Hamiltonian of system can be defined as

1 $$\begin{aligned} H = \sum _{n=1}^N t_1 (A_n ^\dagger B_n+B_n ^\dagger C_n+A_n ^\dagger C_n) +\sum _{n=1}^{N-1} t^{\prime }_2(A_n ^\dagger A_{n+1}+B_n ^\dagger B_{n+1})+\sum _{n=1}^{N-1} t_2(C_n ^\dagger A_{n+1}+C_n ^\dagger B_{n+1})+h.c, \end{aligned}$$

where $X_{n}^\dagger$ ($X_{n}$) is the creation (annihilation) operator on the sublattice $X (= A,B,C)$ of the *n*th unit cell and *N* is the number of unit cell. Also, $t_1=t(1+\delta _1)$ is intra unit cell hopping and $t^{(\prime )}_2=t(1-(+)\delta ^{(\prime )}_2)$ is oblique (horizontal) inter unit cell hopping to the nearest neighbors. $\delta _1$ and $\delta ^{(\prime )}_2$ are some parameters modulating hopping energies. We choose *t* as the unit of energy.

With periodic boundary conditions, *H* is invariant under translations by a unit cell. So, after Fourier transformation, the Hamiltonian can be written in the basis of $\psi ^\dagger _{_k}=(A_k, B_k, C_k)^\dagger$ yielding compact from $H=\sum _k \psi ^\dagger _{_k} {\mathscr {H}}(k)\psi _{_k}$ with

2 $$\begin{aligned} {\mathscr {H}}(k)=\left( \begin{array}{ccc} 2t^{\prime }_2cos(k)&{}t_1&{}t_1+t_2e^{ik}\\ t_1&{}2t^{\prime }_2cos(k)&{}t_1+t_2e^{ik}\\ t_1+t_2e^{-ik}&{}t_1+t_2e^{-ik}&{}0 \end{array}\right) . \end{aligned}$$

Diagonalizing Eq. (2) gets the eigenvalues,

3 $$\begin{aligned} E^0 &= -2t_1+\eta ,\nonumber \\ E^{\pm} &= \frac{1}{2}(\eta \pm \sqrt{\eta ^2 +2(t^2_1+4t^{\prime 2}_2+8t_1t_2cos(k))}), \end{aligned}$$

where $\eta =t_1+2t^{\prime }_2cos(k)$. The possibility of emerging topological phases though gap closing/reopening conditions at the $k_s=(0,\pi )$ would be provided if $E^+=E^-$ leading to

4 $$\begin{aligned} t_1=e^{ik_s}\frac{2}{9}(t^{\prime }_2+4t_2 \pm \frac{\sqrt{-(-2t^{\prime }_2+t_2)^2}}{2}). \end{aligned}$$

Note, Eq. (4) implicates that topological phase transition is possible if the term under the square root is zero, i.e., $t^{\prime }_2=t_2/2$. Under such condition, Eq. (4) indicates phase boundaries distinguishing topologically nontrivial phase from topologically trivial one.

In Fig. 2, we depicted the band structure of system showing closing and reopening of the energy gap between the two bands $E^{+}$ and $E^{-}$ near the topological phase transition point. In Fig. 2a, the gap of system is open and there are no energy states at Fermi energy. So, the system is a NI. With increasing $t_1$, from Fig. 2b, one can see that the gap closes leading to the topological phase transition. At the same time, surprisingly, the band $E^0$ shifts towards the Fermi level and touches it. After topological phase transition, as shown in Fig. 2c, the energy gap between $E^{+}$ and $E^{-}$ is reopened and the band $E^0$ crosses the Fermi level which represents a metallic state. This signals that the whole system will be a conductor in a nontrivial topological phase giving rise a new type of topological phase transition from the NI to the TM phase.

As already mentioned above, if $t^{\prime }_2 \ne t_2/2$ the system does not support any topological phase. Because, in general, Hamiltonian (2) does not have chiral, particle-hole, and/or inversion symmetries. As such, the system provides a trivial phase without any topological edge state in the gap or bulk states under the open boundary conditions. However, if $t^{\prime }_2=t_2/2$ then inversion symmetry in a subspace of the Hilbert space of system would be revived (as will be discussed below) and Eq. (4) reduces to $t_1=t_2e^{ik_s}$ reminiscing the gap closure condition of SSH model^13^. So, hereafter, we set $t^{\prime }_2=t_2/2$ otherwise specified. Note that to satisfy the requirement $t^{\prime }_2=t_2/2$, the hoppings can also be effectively tuned by externally applied periodic fields using Floquet-Bloch theory^14,15^ (for details see^16^).

Furthermore, Hamiltonian ${\mathscr {H}}(k)$ has exchange symmetry with the corresponding exchange operator $\Upsilon$ defined as,

5 $$\begin{aligned} \Upsilon \psi _k=\psi ^\prime _k=\left( \begin{array}{c} B_k\\ A_k\\ C_k \end{array}\right) , \end{aligned}$$

which exchanges the sublattices *A* and *B*. Owing to the presence of such symmetry, $[{\mathscr {H}}(k), \Upsilon ]=0$, and then the Hamiltonian can be block-diagonalized in the basis of $\Upsilon$ under the transformation ${\mathscr {U}}^{-1} {\mathscr {H}}(k){\mathscr {U}}={\mathscr {H}}^{BD}(k)$ yielding

6 $$\begin{aligned} {\mathscr {H}}^{BD}(k)=\left( \begin{array}{cc} h_1&{}0 \\ 0&{}h_2 \end{array} \right) , \end{aligned}$$

where

7 $$\begin{aligned} h_1 &= -2t_1+\eta , \end{aligned}$$

8 $$\begin{aligned} h_2 &= \left( \begin{array}{cc} \eta &{} \sqrt{2}(t_1+t_2e^{ik})\\ \sqrt{2}(t_1+t_2e^{-ik}) &{} 0\\ \end{array}\right) , \end{aligned}$$

and

9 $$\begin{aligned} {\mathscr{U}} & = \frac{1}{\sqrt{2}}\left( \begin{array}{ccc} -1&{}1&{}0 \\ 1&{}1&{}0\\ 0&{}0&{}\sqrt{2} \end{array} \right) . \end{aligned}$$

Note that the obtained bases of exchange operator (9) can be related to a unitary matrix in block-diagonalizing a class of quasi-1D and -2D systems comprised of odd chains^17,18^. In fact, the block-diagonalization (6) splits the Hilbert space of the system into two subspaces so that one can examine their topological properties independently. Interestingly, in a one of the subspaces, the subsystem $h_2$ can be regarded as a generalized SSH model with next-nearest hopping, $t^{\prime }_2$, and on-site potential, $t_1$^19^. Then, the subspace associated with $h_2$ would host topological phases which in combination with the metallic subspace of $h_1$ would make the whole system TM.

To discuss about the symmetry of the subsystem $h_2$ we rewrite its Hamiltonian as

10 $$\begin{aligned} h_2={\mathfrak {h}}_0\sigma _0+\sum _i{\mathfrak {h}}_i\sigma _i, \end{aligned}$$

where $\sigma _0$ is the identity matrix, $\sigma _i$ is the $i(=x,y,z)$ component of the Pauli matrices, and

11 $$\begin{aligned} {\mathfrak {h}}_0 &= {\mathfrak {h}}_z=\eta /2, \end{aligned}$$

12 $$\begin{aligned} {\mathfrak {h}}_x &= \sqrt{2}\eta , \end{aligned}$$

13 $$\begin{aligned} {\mathfrak {h}}_y &= \sqrt{2}t_2sin(k). \end{aligned}$$

It is well-studied that the simultaneous presence of all three components ${\mathfrak {h}}_x$, ${\mathfrak {h}}_y$, and ${\mathfrak {h}}_z$ in 1D two-band Hamiltonians, for instance, SSH model, breaks inversion symmetry and, subsequently destroys topological edge states^20^. But, for our model although ${\mathfrak {h}}_z$ is nonzero, due to the relation ${\mathfrak {h}}_z=\frac{1}{2\sqrt{2}}{\mathfrak {h}}_x$, one can find effective inversion symmetry, $\Pi h_2(k)\Pi =h_2(-k)$, in the subspace of $h_2$ with inversion operator

14 $$\begin{aligned} \Pi &= \frac{1}{3}\left( \begin{array}{cc} 1&{}2\sqrt{2} \\ 2\sqrt{2}&{}-1 \end{array} \right) . \end{aligned}$$

This implies that there exists a hidden inversion symmetry in the system protecting the topological phase. The emergence of inversion-symmetry-protected topological phase can be verified by quantized $\mathbb {Z}$ invariant^21^.

Moreover, the $h_2$ has time-reversal symmetry fulfilling ${\mathscr {T}} h_2(k) {\mathscr {T}}^{-1}=h_2(-k)$ where the time-reversal operator is ${\mathscr {T}}=\sigma _0{\mathscr {K}}$ with ${\mathscr {K}}$ being the complex conjugate operator. Also, the reason for the breaking of chiral symmetry can also be seen from the energy dispersions of the two-band subsystem $h_2$ which are

15 $$\begin{aligned} E^{\pm} ={\mathfrak {h}}_0\pm \sqrt{{\mathfrak {h}}^2_x+{\mathfrak {h}}^2_y+{\mathfrak {h}}^2_z}=\frac{{\mathfrak {h}}_x}{2\sqrt{2}}\pm \sqrt{\frac{9 {\mathfrak {h}}^2_x}{8}+{\mathfrak {h}}^2_y}. \end{aligned}$$

Here, the term ${\mathfrak {h}}_0$ breaks chiral symmetry so, as usual, one may expect that the zero-energy edge states will be shifted by this term. Nevertheless, interestingly, the relation between ${\mathfrak {h}}_0$ and ${\mathfrak {h}}_x$ holds the energy of edge states at zero energy which is in contrast to the previous studies. Therefore, due to ${\mathscr {T}}^2=1$ and $\Pi ^2=1$, according to the generalized periodic table^22–24^, the symmetry class of subsystem $h_2$ is AI with topological index $\mathbb {Z}$. Note that, in our case, the primary periodic table^4^ cannot be applied because it is based on nonspatial symmetries.

The existence of the hidden inversion symmetry guarantees that the eigenvectors of $h_2$ have a well-defined parity at the inversion symmetric momenta $k_s=(0,\pi )$. So, one can define an integer invariant as $\mathbb {Z}=|n_0-n_\pi |$ where $n_0$ and $n_\pi$ denote the number of negative parities at $k_s=0$ and $k_s=\pi$, respectively^21^. The analytical expression of topological invariant $\mathbb {Z}$ for the subsystem $h_2$ can be obtained as (for details see^16^)

16 $$\begin{aligned} \mathbb {Z}= {\left\{ \begin{array}{ll} 0, &{} \text {if} \ sgn(\eta (0))=sgn(\eta (\pi )) \\ 1, &{} \text {if} \ sgn(\eta (0))\ne sgn(\eta (\pi )) \end{array}\right. }, \end{aligned}$$

where *sgn*(*x*) is the Sign function. For $\mathbb {Z} = 1$ a nontrivial topological phase will be revealed in the subspace of $h_2$. In such a situation, due to bulk-edge correspondence, the gapless edge states associated with the nontrivial topological character of bulk states will be appeared at the boundary of the system under open boundary conditions. For $\mathbb {Z} = 0$ the subsystem $h_2$ has a trivial phase.

Generally, combining the two-band subsystem $h_2$ with the single-band subsystem $h_1$ leads to another topological aspect for which the gapped topological bands would coexist with the trivial metallic band resulting in the TM phase^8,9,12,17,18^. This means that when the system has open boundary conditions, in the topologically nontrivial phase of $h_2$, there exist zero-energy edge states near the system boundaries while the subsystem $h_1$ has topologically trivial phase. So, the band structure of the whole system shows an interesting phenomenon that topological edge states of $h_2$ are in the metallic bulk states of $h_1$ instead of being in the system gap as will be shown below.

## Numerical results

To complement the analytical results with numerical ones, we diagonalize the Hamiltonian (1) numerically under open boundary conditions. In order to identify the localized edge states penetrated into extended bulk states under open boundary conditions, in the following, we evaluate the inverse participation ratio (IPR) of states^25^. The IPR for an eigenstate $\psi _E(j)$ in the corresponding eigen energy E is given as

17 $$\begin{aligned} I_E=\frac{Ln \sum _j \vert \psi _E(j) \vert ^4}{Ln 3N}. \end{aligned}$$

If $\psi _E(j)$ is localized, then $I_E=0$, whereas if $\psi _E(j)$ is extended, then $I_E = -1$.

The band structure of system along with its corresponding topological invariant $\mathbb {Z}$ as a function of $t_1$ is plotted in Fig. 3(a) and (b) for the cases $t^{\prime }_2=t_2/2$ and $t^{\prime }_2\ne t_2/2$, respectively. In Fig. 3(a), with establishing the hidden inversion symmetry due to $t^{\prime }_2=t_2/2$, the gap of $h_2$ closes and then reopens at the topological phase transition points such that topological edge states appears. Since the inversion operator can commute with $h_2$, the expectation values of inversion operator at the inversion symmetric momenta k=0 and $\pi$ are either 1 or $-1$. Subsequently, the topological invariant, which is the sum of negative parities, takes the quantized values 0 and 1 for the trivial and nontrivial regimes, respectively. In the nontrivial region, the system hosts the degenerate zero-energy edge states within the bulk states in a width range of $t_1$ indicating the emergence of stable TM phase. Since, in the trivial region, the system is a trivial insulator, as a result, a topological phase transition has occurred between the NI and the stable TM phase. Also, when $t^{\prime }_2\ne t_2/2$, owing to violating the hidden inversion symmetry, as shown in Fig. 3(b), the degeneracy of edge states is destroyed, and depending on values of $t_1$, the system is either NI or trivial metal. Also, the invariant $\mathbb {Z}$ takes continuous values. Because, in this case, the quantum number associated with inversion operator are no longer a good quantum number. As such, the expectation values of inversion operator do not take integer values. In Fig. 3(c), topological phase diagram is shown as functions of $t_1$ and $t^{\prime }_2$. The regions represented by red (gray) colors indicate the nontrivial TM (NI) regime. One can easily see that in addition to the condition $t^{\prime }_2=t_2/2$, when $|t_2|>|t_1|$ the TM phase will be appeared.

**Figure 4:**
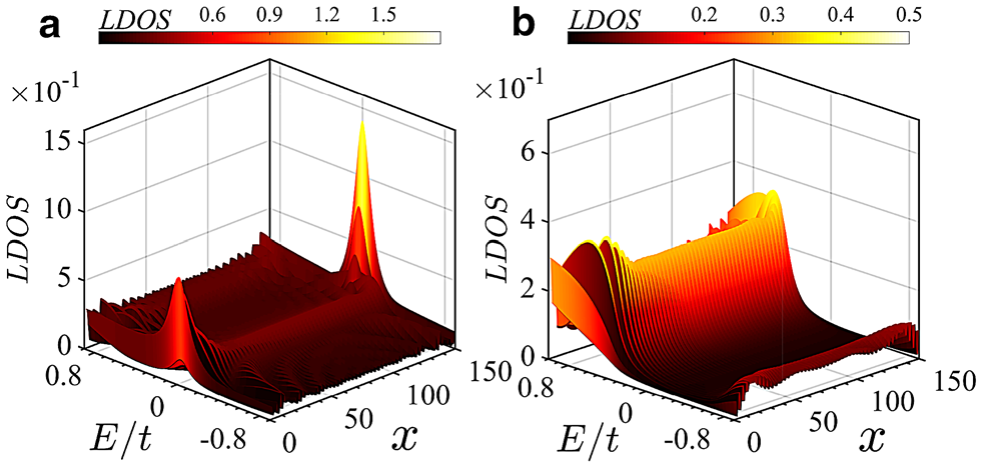
(Color online) Dependence of local density of states on *E* and on unit cell index x in (**a**) the TM phase with $t_1=-0.9t$ and (**b**) the NI phase with $t_1=-1.55t$. Here, $t_2^{\prime }=t_2/2=-0.6t$.

In Fig. 4, the local density of states (LDOS) as functions of energy *E* and unit cell index *x* is displayed. In the TM phase, at zero energy, the localized states are revealed at the ends of the system (see Fig. 4a). One can also see, as mentioned above, these localized edge states coexist with the bulk states. Note that the peak intensities of localized edge states at both ends are not the same. This is because of breaking chiral symmetry. In the NI phase, at Fermi energy, the LDOS vanishes (see Fig. 4b) and the localized edge states disappear.

**Figure 5:**
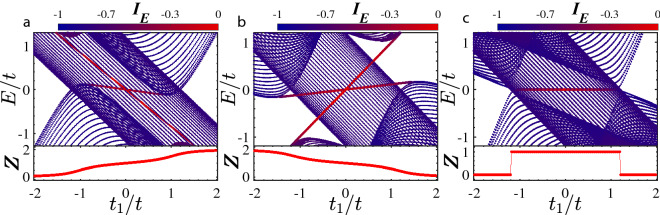
(Color online) Energy spectra and the inverse participation ratio with their relevant topological invariant as a function of $t_1/t$ under open boundary conditions for (**a**) $V_c=0$, $t_c=t_2$ (**b**) $V_c=t_1$, $t_c=0$ (**c**) $V_c=t_1$, $t_c=t_2$. Here, $t_2^{\prime }=t_{2/2}=-0.6t$.

It is well studied that symmetry protected topological states can exist in a system as long as a certain symmetry is preserved. So, the system exhibits the topological edge state in the topological regime in the presence of symmetry. In the present study, to demonstrate that the hidden inversion symmetry has a fundamental role, we examine the stability of the topological phase by considering on-site and off-site Hamiltonians, respectively, given by

18 $$\begin{aligned} H_1 &= \sum _{n=1}^N V_c C_n ^\dagger C_n, \end{aligned}$$

19 $$\begin{aligned} H_2 &= \sum _{n=1}^{N-1} t_c C_n ^\dagger C_{n+1} + h.c, \end{aligned}$$

breaking the inversion symmetry of the system. Here, $V_c$ and $t_c$ are the strength of on-site and off-side potentials. Such Hamiltonians are chosen such that the condition $t_2^{\prime }=t_2/2$ remains intact. At first, we add each of the above Hamiltonians to Eq. (1) separately and calculate the band structure under open boundary conditions. The band structure and the corresponding topological invariant $\mathbb {Z}$ as a function of $t_1$ are depicted in Fig. 5(a) and (b). Although one may expect that due to fulfilling the condition $t_2^{\prime }=t_2/2$, the system would host topological edge states, but either of the two perturbations breaks the inversion symmetry, resulting in lifting the degeneracy of topological edge states and in destroying the TM phase. On the other hand, if we add both $H_1$ and $H_2$ to the main Hamiltonian (1) simultaneously for specific strength $V_c=t_1$ and $t_c=t_2$, the hidden inversion symmetry will be restored and, as shown in Fig. 5(c), the system hosts the topological edge states at zero energy with nontrivial value of $\mathbb {Z}$.

## Summary and discussion

We introduced a quasi-1D model that presents a new topological phase transition between the TM phase and NI. In such topological phase transition, in addition to bandgap closing-reopening, another band crosses the Fermi level. The Hamiltonian of system can be block-diagonalized into two subsystems in the presence of exchange symmetry. One of these subsystems can be regarded as the generalized SSH model hosting topological edge states when the band of other subsystem passes the Fermi level resulting in the TM phase. Finally, the edge states created in the absence of chiral or particle-hole symmetry and protected by the hidden inversion symmetry. By breaking the hidden inversion symmetry, depending on the parameter, the system has either NI or metallic state with nondegenerate trivial edge states.

Experimentally, our model can be realized by coupled acoustic resonators^18^, topolectrical circuits^26^, optical lattices^27^, photonic crystals^28,29^, and mechanical systems^30–34^. Using cold atoms, it is possible to simulate quasi-1D chains^35^ and to reveal the topological features employing density and momentum-distribution measurements^36^. Also, using spatially resolved radio-frequency spectroscopy^37^, the topological edge states can be probed by the LDOS. Furthermore, using edge state transport in topological states of matter, one can distinguish between topologically trivial and nontrivial edge states^38,39^.

## Supplementary information

Supplementary Information.
