# Supplementary material for: Topological phase transition between a normal insulator and a topological metal state in a quasi-one-dimensional system
Source: Sci Rep. 2021 Jun 21;11:12966. doi: 10.1038/s41598-021-92390-x (PMC8217258; doi:10.1038/s41598-021-92390-x)
Supplement: Supplementary file 1 — Supplementary Information. [file 41598_2021_92390_MOESM1_ESM.pdf]

Supplementary Material for "Topological phase transition between normal insulator and topological metal state in a quasi-one-dimensional system"

Milad Jangjan and Mir Vahid Hosseini
Department of Physics, Faculty of Science, University of Zanjan, Zanjan 45371-38791, Iran

In this supplementary material, we discuss how using the Floquet formalism that takes into account a dynamic of the irradiated quasi-1D system, one can establish topological phase dynamically. Also, we will derive analytical expressions for the $\mathbb{Z}$ invariant of the system.

FLOQUET ENGINEERING OF HOPPING

In this section, using the Floquet-Bloch theory [1, 2], we show how the hoppings can be modulated appropriately to satisfy the requirements discussed in the main text, exhibiting the TM phase dynamically. We consider that our system is irradiated by a time-periodic polarized electromagnetic field [3], as shown in Fig. S1. We assume a large frequency for the driving field so that the time-periodically driven system can be described by an effective time-independent Hamiltonian [4], called a Floquet Hamiltonian.

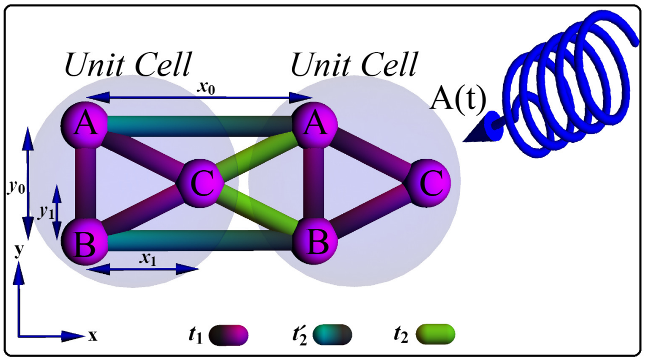

FIG. S1. (Color online) Schematic geometry of the quasi-1D lattice illuminated by time-periodic polarized laser beam. x_0 and y_0 are the length and width of unit cell, respectively. x_1 and y_1 are horizontal and vertical spacings of sublattice C from B, respectively.

To manifest topological edge states in the system dynamically [5], we apply an elliptically polarized beam on the system. Mathematically, the effect of beam with periodic time-dependent electric field $\mathbf{E}(t) = -\partial_t \mathbf{A}(t)$ with vector potential $\mathbf{A}(t) = (A_x sin\omega t, A_y sin(\omega t + \phi))$ on the system can be incorporated by Pierles substitution through the change the hoppings as

$$t_{ij} \longrightarrow t_{ij} e^{-i\frac{e}{\hbar c}\int_{R_i}^{R_j} \mathbf{A}(t)\cdot d\mathbf{r}}, \tag{S1}$$

where c is light speed, and e is electron charge. We take $\hbar = 1$ and $\frac{e}{c} = 1$ hereafter. It is obvious that the Hamiltonian of a system that is exposed to a polarized laser field change to a time-dependent Hamiltonian. In a case where the Hamiltonian is time-dependent $H(t)$, the time-evolution of the system is governed by the Schrödinger equation $H(t)|\Psi(t)\rangle = i\partial_t|\Psi(t)\rangle$ which one can expand the solution as $|\Psi_\beta(t)\rangle = e^{i\epsilon_\beta t}|\psi_\beta(t)\rangle$ where ϵ_β is the quasi-energy and $|\psi_\beta\rangle$ is the βth Floquet state. For a time-periodic system, $H(t) = H(t+T)$, the Schrödinger equation leads to $H_F|\psi_\beta\rangle = \epsilon_\beta|\psi_\beta\rangle$ where $H_F = H(t) - i\partial_t$ is the Floquet Hamiltonian with the matrix elements

$$H_F^{\beta\beta'} = \frac{1}{T}\int_0^T H(t) e^{i(\beta-\beta')\omega t} dt - \beta\omega\delta_{\beta\beta'}. \tag{S2}$$

Here, β and β' stand for Floquet index and the period $T = \frac{2\pi}{\omega}$ is determined by the driving frequency ω.

So, by substituting Eq. (S1) in the Hamiltonian (1) of the main text and using Eq. (S2), the zeroth-order Floquet Hamiltonian ($\beta = \beta' = 0$) in the high-frequency regime can be rewritten as

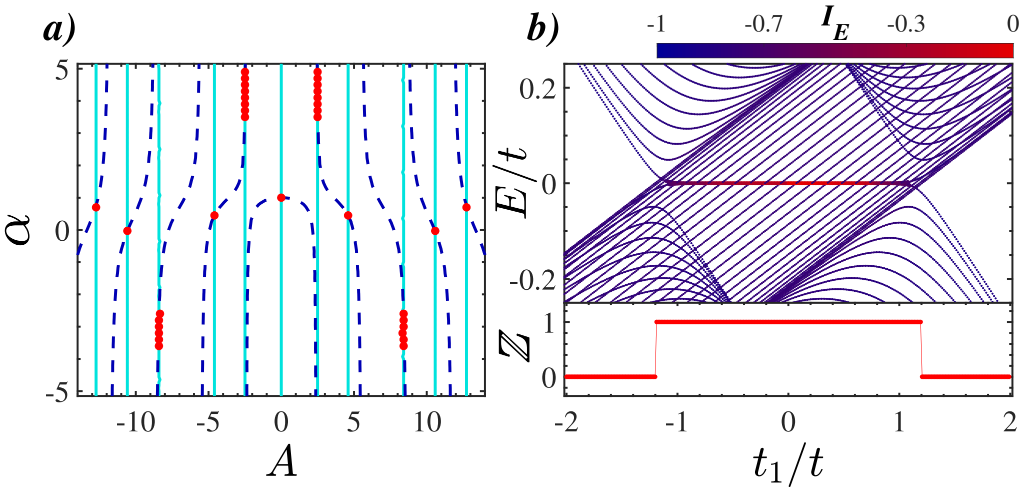

FIG. S2. (Color online) (a) Acceptable solutions (red dots) are obtained from the intersections of Eqs. (S5) (solid lines) and Eq. (S6) (dashed lines) restoring the hidden inversion symmetry. (b) Energy spectra and the relevant topological invariant as a function of t_1 for $A_x = A_y/4 = A = 2.495$, $x_0 = 1$, $y_0 = 0.5$, $t_2 = 1.2t$, and $\alpha = 3.93$.

$$\mathcal{H}_F(k) = \begin{pmatrix} 2t'_2 J_{aa} cos(k) & t_1 J_{ab} & t_1 J_{ac} + t_2 J'_{ac} e^{ik} \\ t_1 J_{ab} & 2t'_2 J_{aa} cos(k) & t_1 J_{bc} + t_2 J'_{bc} e^{ik} \\ t_1 J_{ac} + t_2 J'_{ac} e^{-ik} & t_1 J_{bc} + t_2 J'_{bc} & 0 \end{pmatrix}, \tag{S3}$$

where

$$\begin{aligned} J_{ab} &= J_0[A_y y_0], \\ J_{ac} &= J_0[\sqrt{(A_x(x_1))^2 + (A_y(y_0 - y_1))^2 - 2A_x A_y (x_1)(y_0 - y_1) cos(\phi)}], \\ J_{bc} &= J_0[\sqrt{(A_x(x_1))^2 + (A_y y_1)^2 + 2A_x A_y (x_1) y_1 cos(\phi)}], \\ J'_{ac} &= J_0[\sqrt{(A_x(x_0 - x_1))^2 + (A_y(y_0 - y_1))^2 + 2A_x A_y (x_0 - x_1)(y_0 - y_1) cos(\phi)}], \\ J'_{bc} &= J_0[\sqrt{(A_x(x_0 - x_1))^2 + (A_y y_1)^2 - 2A_x A_y (x_0 - x_1) y_1 cos(\phi)}], \\ J_{aa} &= J_0[A_x x_0]. \end{aligned} \tag{S4}$$

with $J_n(x) = \frac{1}{T}\int_0^T e^{ixsin(\omega t) - in\omega t} dt$ is the first kind Bessel function of order n. Also, the x_0 and y_0 are the lattice constants and the x_1 (y_1) is the horizontal (vertical) length between C and B sublattices in each unit cell. For simplicity, we set $\phi = \pi/2$, $x_1 = x_0/2$, and $y_1 = y_0/2$ so that $J_{ac} = J_{bc} = J'_{ac} = J'_{bc}$. Thus, the matrix structure of (S3) becomes similar to Eq. (2) of the main text, if

$$J_{ab} = J_{ac}. \tag{S5}$$

Furthermore, in order to establish the hidden inversion symmetry one must have

$$2t'_2 J_{aa} = t_2 J'_{bc}. \tag{S6}$$

We assume $t'_2 = \alpha t_2/2$ with $\alpha \neq 1$ imposing the hidden inversion symmetry to be broken in the static case. We solved (S5) and (S6) graphically, as shown in Fig. S2(a), to get possible field amplitudes and α restoring the hidden inversion symmetry dynamically. The solutions of conditions (S5) and (S6) are represented by solid and dashed lines for polarization vector $(A_x, A_y) = (A, A/4)$. The intersections of the solid and dashed lines, indicated by red dots, provide the values of parameter α and field amplitudes for which both (S5) and (S6) are satisfied simultaneously. In Fig. S2(b), the band structure of system and its topological invariant $\mathbb{Z}$ versus t_1 are shown for the situation where the hidden inversion symmetry is broken in the static case, i.e., $\alpha \neq 1$. One can see that for certain values of α and (A_x, A_y) fulfilling both (S5) and (S6), the topological edge states would be appeared in a space of parameters that was not topologically nontrivial in the static case.

DERIVATION OF $\mathbb{Z}$

After diagonalizing the subsystem h_2, the eigenstate of lower (filled) band can be obtained as

$$\psi(k) = \frac{\sqrt{8 + |1 - \frac{3\eta(k)}{|\eta(k)|}|^2}}{2\sqrt{2}} \begin{pmatrix} \frac{\eta(k) - 3|\eta(k)|}{2\sqrt{2}\eta(k)} \\ 1 \end{pmatrix}. \tag{S7}$$

So, the expectation value of inversion operator Π at $k_s = (0, \pi)$ yields

$$\langle \Pi \rangle_{k_s=0} = \langle \psi(0)|\Pi|\psi(0)\rangle = \begin{cases} -1, & \text{if } \eta(0) < 0 \\ 1, & \text{if } \eta(0) > 0 \end{cases}, \tag{S8}$$

$$\langle \Pi \rangle_{k_s=\pi} = \langle \psi(\pi)|\Pi|\psi(\pi)\rangle = \begin{cases} -1, & \text{if } \eta(\pi) < 0 \\ 1, & \text{if } \eta(\pi) > 0 \end{cases}. \tag{S9}$$

Therefore, the number of negative parities at $k_s = 0$ and $k_s = \pi$, i.e., n_0 and n_π, respectively, are

$$n_0 = \begin{cases} 1, & \text{if } \eta(0) < 0 \\ 0, & \text{if } \eta(0) > 0 \end{cases}, \tag{S10}$$

$$n_\pi = \begin{cases} 1, & \text{if } \eta(\pi) < 0 \\ 0, & \text{if } \eta(\pi) > 0 \end{cases}. \tag{S11}$$

Summarizing Eqs. (S10) and (S11), one obtains $\mathbb{Z}$ as Eq. (16) of the main text.

[1] J. H. Shirley, Phys. Rev. **138**, B979 (1965).
[2] H. Sambe, Phys. Rev. A **7**, 2203 (1973).
[3] N. Lindner, G. Refael, and V. Galitski, Nat. Phys. **7**, 490 (2010).
[4] F. Harper, R. Roy, M. S. Rudner, and S. L. Sondhi, Annu. Rev. Condens. Matter Phys. **11**, 345 (2020).
[5] A. Gómez-León and G. Platero, Phys. Rev. Lett. **110**, 200403 (2013).
